# Supplementary material for: Concurrent Validation of MI-CAT(V), a Clinical Metrology Instrument for Veterinarians Assessing Osteoarthritis Pain in Cats, through Testing for Firocoxib Analgesic Efficacy in a Prospective, Randomized, Controlled, and Blinded Study
Source: Animals (Basel). 2024 Feb 24;14(5):711. doi: 10.3390/ani14050711 (PMC10931368; doi:10.3390/ani14050711)
Supplement: Supplementary file 1 [file animals-14-00711-s001.zip › animals-2826743-supplementary.pdf]

Supplementary materials

Figure S1. MI-CAT(V) refined version composed of four sub-sections.

Montreal Instrument for Cat Arthritis Testing, for Veterinarian use [MI-CAT(V)]

Evaluation Procedure

Perform the evaluation by observing the cat from a distance. Place the cat on the floor and observe its undisturbed behavior, posture, and movements.

Stimulate walking, running, jumping, playing, *etc*. Toss treats or use toys (*e.g.* cotton ball, strings) for it to chase. Call, gesture enticingly or pet the cat to encourage it to approach the evaluator.

Criterion 1. Assess **Body Posture** both with the cat standing still and during locomotion.

Criterion 2. Observe **Gait** from all perspectives (including from above). If needed, use encouragement to stimulate the cat to walk, run and turn.

Do not physically push or pull the cat, nor guide it by a leash or collar, nor startle it. These will interfere with natural movements.

Criterion 3. Assess **Obstacles** by encouraging the cat to pass under a low and adjustable overhead obstacle of approx. its elbow height (*e.g.*, rungs of a cart or gurney…height around 12-17 cm). Encourage the cat to jump down using a raised horizontal surface (*e.g.*, bench, shelf, chair, low table … height around 76 cm). Jump height measurement is a good discriminatory factor in discomfort as severely affected cats will lean on the vertical support with the thoracic limbs in the hope to reduce the jump size. A rescue stair could be available as alternative to give the choice to the osteoarthritic cat to either jump down or use the rescue stair.

Criterion 4. Complete the **Global Distance Exam** based on your global subjective impression.

NOTE: Cats with osteoarthritis do not necessarily demonstrate all of the abnormalities described below.

Scoring of Abnormality

Each item (criterion) should be scored in terms of **Degree of Alteration** as indicated for each criterion.
If an item cannot be evaluated, its maximum score will be deducted from the criterion maximum score.

**Assessment Criteria**	**Degree of Alteration**			**Score**
**1. Body Posture**	Normal	Mild	(in intensity or duration) Clear/Obvious	
A. Stands with hind limbs held far forward under body	0	1	3	
B. Increased forelimb flexion	0	1	3	
C. Increased hind limb flexion	0	1	3	
D. Hind limb asymmetry (right to left)	0	1	3	
			**Total score** (add items A to D)	

Assessment Criteria	Degree of Alteration					Score
**2. Gait**	Normal	Possible	(or doubtful)	Clear/Obvious		
A. Appears to move slowly	0	1		3		
B. Forelimb stiffness/lameness	0	1		3		
C. Hind limb stiffness/lameness	0	1		3		
D. Forelimb joints – reduced range of motion	0	1		3		
E. Hind limb joints – reduced range of motion	0	1		3		
F. Lateral movements of spine at walk	0	1		3		
**Total score** (add items A to F)						
**Maximum score = • •** (*subtract • for each unscored item*)						
**3. Obstacles**						
A. Difficulty passing under an overhead obstacle	0 (easy)	1 (not so easy)	2 (hesitant)	3 (difficult)	4 (no attempt)	
Speed						
Willingness						
Scrape loudness						
B. Front feet land heavily (audibly/visibly) when jumping down	0 (not perceptible)		1 (noisy)	3 (loud)	4 (no attempt)	Preferential use of rescue stair 4
Hind feet land heavily (audibly/visibly) when jumping down	0 (not perceptible)		1 (noisy)	3 (loud)	4 (no attempt)	Preferential use of rescue stair 4
**Total score** (add items A to B)						
**Maximum score = • •** (*subtract • for each unscored item*)						

4. Global Distance Examination

No mobility impairment									Worst possible OA-related mobility impairment
☐	☐	☐	☐	☐	☐	☐	☐	☐	☐
1	2	3	4	5	6	7	8	9	10

	Score
**Score**	
**Maximum score = 10**	
**(A) MI-CAT(V) Total score** (add scores from criteria 1 to 4)	
**(B) Maximum scale score = • •** (*subtract as needed for unanswered items*)	
**Maximum score = • •** (*subtract • for each unscored item*)	

Table S1. Descriptive statistics of the MI-CAT(V) clusters.

	**Mild** MI-CAT(V) score ≤ 20%			**Moderate** MI-CAT(V) score between 21-35%			**Severe** MI-CAT(V) score > 35%		
	Baseline	Treatment	Recovery	Baseline	Treatment	Recovery	Baseline	Treatment	Recovery
MI-CAT(V) total	13.92 (3.33-20.00)	15.83 (6.67-41.67)	21.33 (13.33-33.33)	27.59 (25.83-30.00)	18.33 (8.33-31.67)	23.15 (11.67-45.00)	40.00 (36.67-45.00)	18.33 (16.67-21.67)	27.92 (18.33-40.00)
Body posture	1.70 (0.00-3.00)	1.20 (0.00-5.00)	2.10 (1.00-4.00)	3.00 (2.00-5.00)	2.33 (1.00-5.00)	2.67 (1.00-6.00)	5.00 (3.00-6.00)	1.50 (1.00-3.00)	2.75 (2.00-3.00)
Gait	2.20 (1.00-4.00)	2.10 (0.00-6.00)	3.70 (1.00-6.00)	4.67 (3.00-6.00)	2.11 (0.00-5.00)	3.89 (2.00-9.00)	7.00 (4.00-9.00)	2.50 (2.00-3.00)	4.25 (1.00-7.00)
Global Dist. Exam.	2.30 (1.00-3.00)	3.00 (2.00-6.00)	3.60 (2.00-5.00)	3.89 (3.00-4.00)	3.11 (2.00-5.00)	3.56 (2.00-6.00)	4.75 (4.00-6.00)	3.50 (3.00-4.00)	4.25 (3.00-5.00)
Obstacles	2.30 (0.00-6.00)	3.20 (1.00-8.00)	3.80 (1.00-6.00)	5.89 (4.00-7.00)	3.44 (1.00-7.00)	4.44 (1.00-9.00)	7.75 (7.00-9.00)	3.50 (2.00-5.00)	3.75 (2.00-6.00)
Path – Velocity	1.53 (0.92-2.48)	1.67 (1.51-2.13)	1.52 (1.20-2.00)	1.52 (0.53-2.62)	1.58 (0.68-1.85)	1.62 (0.12-2.20)	0.97 (0.46-1.62)	1.21 (0.69-1.77)	1.31 (0.54-1.87)
Path – Time of completion	36.30 (27.00-61.00)	30.80 (25.00-42.00)	33.30 (28.00-44.00)	37.06 (29.00-58.00)	35.33 (30.00-50.00)	44.00 (27.00-154.00)	59.75 (30.00-106)	35.00 (29.00-38.00)	42.25 (28.00-57.00)
Path – Thoracic PVF	0.53 (0.45-0.75)	0.51 (0.39-0.74)	0.51 (0.45-0.72)	0.52 (0.45-0.62)	0.52 (0.40-0.65)	0.51 (0.45-0.65)	0.46 (0.40-0.51)	0.48 (0.48-0.49)	0.47 (0.41-0.55)
NAM	35397.45 (20674.00-67582.00)	34702.90 (17751.00-57369.00)	37906.10 (12807.00-85844.00)	25506.00 (8683.00-52233.00)	31327.00 (10627.00-60854.00)	29234.75 (10508.00-57072.00)	25849.00 (17713.00-33593.00)	30615.25 (18889.00-44171.00)	22243.75 (15260.00-39594.00)
Stairs-Finish Line Up	5.70 (2.00-9.00)	6.40 (4.00-9.00)	6.20 (3.00-9.00)	6.11 (2.00-9.00)	7.00 (2.00-9.00)	8.13 (7.00-10.00)	5.88 (3.00-8.00)	7.50 (7.00-9.00)	6.75 (5.00-9.00)
**Mean (Min-Max)**									

Figure S2. MI-CAT(V) Manual of Use.

Evaluator: ____________ Cat: ____________ Date: ___________

MI-CAT(V) scoring sheet

Montreal Instrument for Cat Arthritis Testing, for Veterinarian use

Assessment Criteria / Degree of Alteration

1. Body Posture

	Normal	Mild	Severe/Obvious
A) Hindlimb placement	0	1	3
B) Forelimb flexion	0	1	3
C) Hindlimb flexion	0	1	3
D) Hindlimb weight bearing	0	1	3

2. Gait

	Normal	Mild	Severe/Obvious
A) Moves slowly with short uneven strides	0	1	3
B) Forelimb stiffness	0	1	3
C) Hindlimb stiffness	0	1	3
D) Forelimb range of motion	0	1	3
E) Hindlimb range of motion	0	1	3
F) Lateral spine movement	0	1	3

3. Obstacles

A) Overhead obstacle at elbow height of cat

					(no attempt)
Speed	0 (run)	1 (fast walk)	2 (slow walk)	3 (crawl)	4
Willingness	0 (immediate)	1 (1-2s pause)	2 (2-5s pause)	3 (>5s pause)	4
Scrape loudness	0 (no sound)	1 (slight scrape)	2 (moderate scrape)	3 (loud scrape)	4

[Assess when cat is chasing a highly desired object (e.g. treat) when the overhead obstacle]

B) Jumping from raised obstacle

	(barely perceptible)	(perceptible)	(loud)	(no attempt)
Front feet land heavily	0	1	3	4
Hind feet land heavily	0	1	3	4

4. Global distance exam

No mobility impairment — Worst possible mobility

1 — 2 — 3 — 4 — 5 — 6 — 7 — 8 — 9 — 10

MI-CAT(V) total score = sum of all items

1. Body Posture

Observe when cat standing still. DO NOT assess when cat moving or rubbing itself against objects or evaluators, playing or any other behaviours that may affect scoring. If one side (left or right) seems differently affected, score the more severely affected side.

	Normal<br>Score: 0	Mild<br>Score: 1	Severe/obvious<br>Score: 3

1A) Hindlimb placement

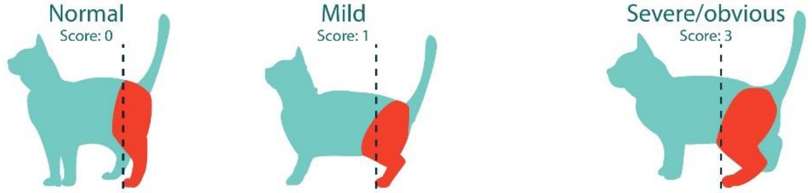

Hindlimbs straight | Hindlimbs bent and forward; **toes approaching middle of belly** | Hindlimbs bent and forward; **toes almost at middle of belly**

1B) Forelimb flexion

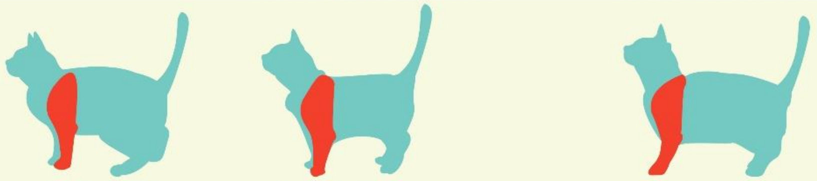

Forelimb straight | Forelimb slightly bent | Forelimb obviously bent

1C) Hindlimb flexion

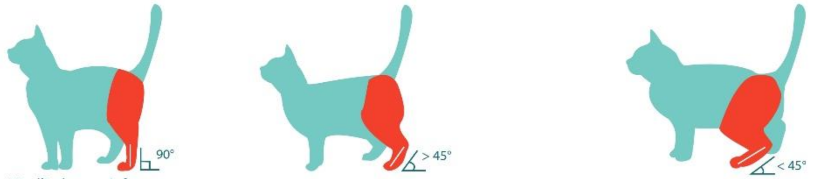

Hindlimbs **straight** approx 90° to the floor | Hindlimbs **slightly bent** approx > 45° formed between heel and floor | Hindlimbs bent, **heel close to ground** approx. <45° formed between heel and floor

Designed by: VSY Leung - © GREPAQ 2020/03 - Update 2023/12

1. Body Posture

Observe when cat standing still. DO NOT assess when cat moving or rubbing itself against objects or evaluators, playing or any other behaviours that may affect scoring. If one side (left or right) seems differently affected, score the more severely affected side.

	Normal<br>Score: 0	Mild<br>Score: 1	Severe/obvious<br>Score: 3

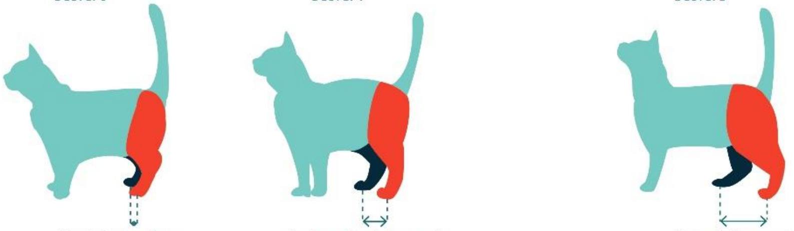

Feet side by side | Feet moderately apart | Feet widely apart

1D) Hindlimb weight bearing

-observe if body weight is evenly distributed on both hindfeet

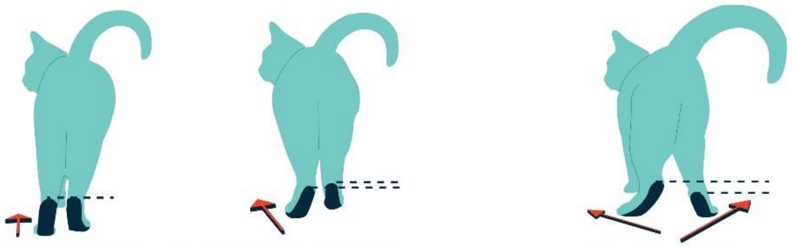

Feet **facing straight** forward and **heels at similar heights** | Feet **facing to side** slightly and/or **heels at slightly different heights** | Feet **facing to side** and/or **heels at different heights**

Designed by: VSY Leung - © GREPAQ 2020/03 - Update 2023/12

2. Gait

Observe when cat is walking. DO NOT assess when cat is running (except for 2A), rubbing itself against objects or evaluators, playing or any other behaviours that may affect scoring. If one side (left or right) seems differently affected, score the more severely affected side.

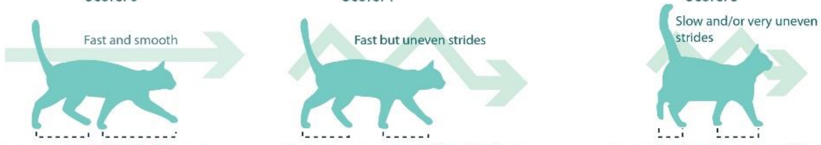

	Normal<br>Score: 0	Mild<br>Score: 1	Severe/obvious<br>Score: 3
2A) Moves slowly with short uneven strides<br>-assess speed, quality of movement and overall willingness to move in general or chase desired object (*e.g.* treats)	Cat runs or trots **quickly** with **long smooth strides**; **high motivation** to move	Cat runs or trots **quickly** with **short uneven strides**; **high motivation** to move	Cat walks **slowly or clumsily**; **low motivation** to move

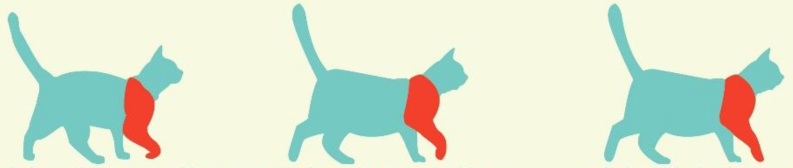

	Normal	Mild	Severe/obvious
2B) Forelimb stiffness	Forelimb muscles contract to lift paw and **metacarpal bends easily**	Forelimb muscles contract to lift paw and **metacarpal bends slightly**	Forelimb muscles barely contract when lifting paw and **metacarpal barely bends**

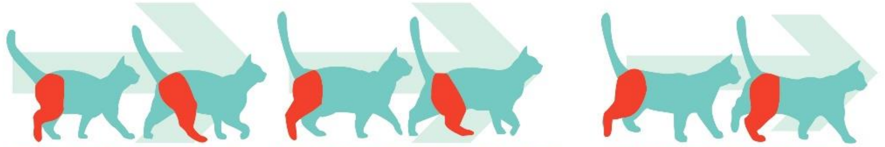

	Normal	Mild	Severe/obvious
2C) Hindlimb stiffness	Hindlimb stretches forward when taking a step; **movement appears fluid**	Hindlimb stretches forward slightly when taking a step; **movement appears slightly stiff**	Angle between tibia and metatarsus stays unchanged; **cat appears to be waddling and movement is very stiff**

Designed by: VSY Leung - © GREPAQ 2020/03 - Update 2023/12

2. Gait

Observe when cat is walking. DO NOT assess when cat is running (except for 2A), rubbing itself against objects or evaluators, playing or any other behaviours that may affect scoring. If one side (left or right) seems differently affected, score the more severely affected side.

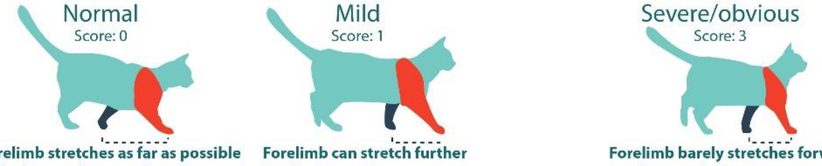

	Normal<br>Score: 0	Mild<br>Score: 1	Severe/obvious<br>Score: 3
2D) Forelimb range of motion	**Forelimb stretches as far as possible**<br>Wide distance between forepaws	**Forelimb can stretch further**<br>Smaller steps taken	**Forelimb barely stretches forward**<br>Small steps taken

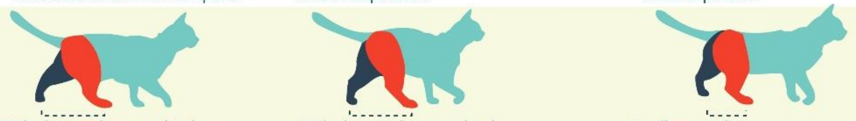

	Normal	Mild	Severe/obvious
2E) Hindlimb range of motion	Wide distance between hindpaws	Wide distance between hindpaws	Small steps taken<br>Small distance between hindpaws

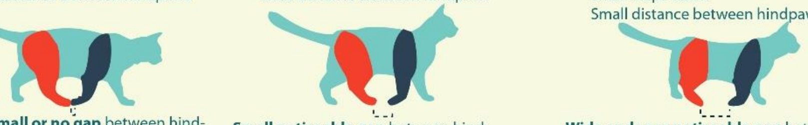

	Normal	Mild	Severe/obvious
2E) Hindlimb range of motion	**Very small or no gap** between hind- and forepaw, **hindpaw almost steps into place of forepaw**	**Small noticeable gap** between hind- and forepaw, **hindpaw does not step into place of forepaw**	**Wide and very noticeable gap** between hind- and forepaw, **hindpaw steps a large distance from where forepaw was**

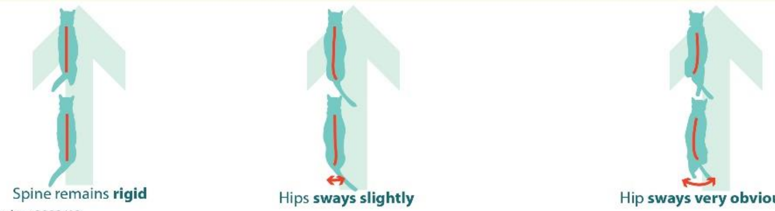

	Normal	Mild	Severe/obvious
2F) Lateral spine movement<br>-assess from behind while cat in motion	Spine remains **rigid**	Hips **sways slightly**	Hip **sways very obviously**

Designed by: VSY Leung - © GREPAQ 2020/03 - Update 2023/12

3. Obstacles

Encourage cat to pass under overhead obstacle (cat's elbow height) and jump from table height (approx. 76 cm)
DO NOT physically push or pull cat by leash or collar or startle it. Use of treats or food is recommended.

3A) Difficulty passing under obstacle

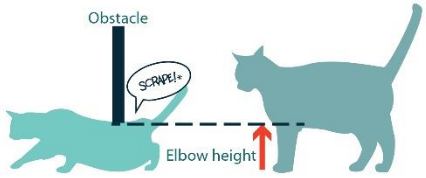

	Score 0	Score 1	Score 2	Score 3
**Speed:**	Run	Fast walk	Slow walk	Crawl
**Willingness:**	Immediate	1-2s pause	2-5s pause	>5s pause
**Scrape loudness*:**	No sound	Slight	Moderate	Loud

Right after throwing treat under overhead obstacle:

- Speed = pace used to chase after treat
- Willingness = reaction time to chase after treat and go under obstacle
- Scrape loudness = sound of cat's back sccratching against obstacle as it goes under

Give score of 4 to each item if cat refuses to attempt

3B) Jumping from raised obstacle

*Assess loudness of landing for front and back feet:
not perceptible (score 0), perceptible (score 1), loud (score 3), did not try (score 4)

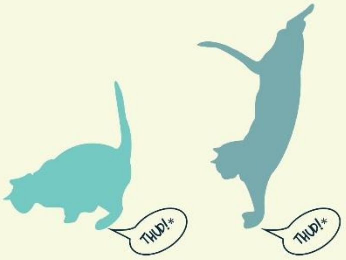

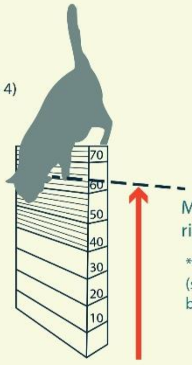

Measure height of cat's paw from ground right before jump**

**Recording and reviewing video footage (slow-motion feature) from a smart phone may be helpful.

Designed by: VSY Leung - © GREPAQ 2020/03 - Update 2023/12
